# Monoamine Oxidase A is Required for Rapid Dendritic Remodeling in Response to Stress

**DOI:** 10.1093/ijnp/pyv035

**Published:** 2015-04-24

**Authors:** Sean C Godar, Marco Bortolato, Sarah E Richards, Felix G Li, Kevin Chen, Cara L Wellman, Jean C Shih

**Affiliations:** Department of Pharmacology and Pharmaceutical Sciences, School of Pharmacy, University of Southern California, Los Angeles, CA (Drs Godar, Chen, and Shih and Mr Li); Department of Cell and Neurobiology, University of Southern California, Los Angeles, CA (Dr Shih); Department of Pharmacology and Toxicology, University of Kansas, Lawrence, KS (Drs Godar and Bortolato); Consortium for Translational Research on Aggression and Drug Abuse (ConTRADA), University of Kansas, Lawrence, KS, (Drs Godar and Bortolato); Department of Psychological & Brain Sciences and Program in Neuroscience, Indiana University, Bloomington, IN (Ms Richards and Dr Wellman)

**Keywords:** Basolateral amygdala, monoamine oxidase A, orbitofrontal cortex, stress

## Abstract

**Background:**

Acute stress triggers transient alterations in the synaptic release and metabolism of brain monoamine neurotransmitters. These rapid changes are essential to activate neuroplastic processes aimed at the appraisal of the stressor and enactment of commensurate defensive behaviors. Threat evaluation has been recently associated with the dendritic morphology of pyramidal cells in the orbitofrontal cortex (OFC) and basolateral amygdala (BLA); thus, we examined the rapid effects of restraint stress on anxiety-like behavior and dendritic morphology in the BLA and OFC of mice. Furthermore, we tested whether these processes may be affected by deficiency of monoamine oxidase A (MAO-A), the primary enzyme catalyzing monoamine metabolism.

**Methods:**

Following a short-term (1–4h) restraint schedule, MAO-A knockout (KO) and wild-type (WT) mice were sacrificed, and histological analyses of dendrites in pyramidal neurons of the BLA and OFC of the animals were performed. Anxiety-like behaviors were examined in a separate cohort of animals subjected to the same experimental conditions.

**Results:**

In WT mice, short-term restraint stress significantly enhanced anxiety-like responses, as well as a time-dependent proliferation of apical (but not basilar) dendrites of the OFC neurons; conversely, a retraction in BLA dendrites was observed. None of these behavioral and morphological changes were observed in MAO-A KO mice.

**Conclusions:**

These findings suggest that acute stress induces anxiety-like responses by affecting rapid dendritic remodeling in the pyramidal cells of OFC and BLA; furthermore, our data show that MAO-A and monoamine metabolism are required for these phenomena.

## Introduction

The neurobehavioral response to acute stress encompasses multiple adaptive processes aimed at appraising the degree of threat or challenge posed by the stressor, and enacting an adequate allostatic reaction to cope with it (McEwen and Wingfield, 2003). These phenomena are accompanied by variations in the release and turnover of monoamine neurotransmitters (including serotonin, dopamine, and norepinephrine) across corticolimbic regions, which result in emotional responses such as heightened anxiety and threat responsiveness (De Boer and Koolhaas 2003; Flugge et al., 2004). The relation between monoamine signaling and stress response is exemplified by the behavioral phenotypes associated with the inactivation of monoamine oxidase (MAO) A, the primary enzyme catalyzing the brain metabolism of serotonin, dopamine, and norepinephrine (Shih et al., 1999; Bortolato et al., 2008). In particular, several studies have shown that MAO-A knockout (KO) mice exhibit blunted and maladaptive responses to stressful contingencies (Kim et al., 1997; Popova et al., 2006; Godar et al., 2011).

Recent findings have documented that the anxiogenic effects of stress also reflect cytoarchitectural alterations and neuroplastic remodeling of dendritic arbors in the output neurons of the corticolimbic circuits (Izquierdo et al., 2006; Mitra and Sapolsky, 2008; Maroun et al., 2013). In particular, changes in dendritic arborization of specific regions, such as the orbitofrontal cortex (OFC) and basolateral amygdala (BLA), have been associated with anxiety-like behaviors, regulation of threat responsiveness, and behavioral adaptation (Fuchs et al., 2006; Dias-Ferreira et al., 2009; Walton et al., 2011; McEwen et al., 2012). Furthermore, the connectivity of these two regions may be instrumental for the reappraisal of stress-related cues and the regulation of emotional responses to stress (Gold et al., 2014; Wheelock et al., 2014).

While cogent evidence has documented that acute stress can exert a long-standing impact on dendritic morphology, the temporal dynamics of this relation remain unclear; for instance, although previous work has shown that acute stress has profound effects on dendritic remodeling of the neurons in the BLA, these changes were only assessed three days after the cessation of the stress (Izquierdo et al., 2006; Maroun et al., 2013), but not at shorter time intervals.

In light of this background, here we studied whether the anxiogenic effects of acute restraint stress (ARS) may be accompanied by rapid (1–4h) changes in the dendritic organization of OFC and BLA neurons. We then investigated whether these phenotypes may be related to monoaminergic neurotransmission by comparing them with the behavioral and morphological effects displayed by MAO-A KO mice subjected to the same stressful conditions.

## Material and Methods

### Animal Husbandry

We used 3–5 month old experimentally-naïve male 129S6 mice weighing 26–32g. MAO-A^A863T^ KO mice (MAO-A KO) were generated and genotyped as previously described (Godar et al., 2011). MAO-A KO sires and heterozygous dams were crossed to generate MAO-A KO and wild-type (WT) male littermates. Animals were housed in group cages with *ad libitum* access to food and water. The room was maintained at 22°C, on a 12h:12h light/dark cycle. To avoid potential carry-over effects, each animal was used only once throughout the study. Litter effects were minimized by using mice from at least six different litters in each behavioral test. Behaviors were tested between the hours of 09:00 to 15:00 on a 06:00 to 18:00 on-off light cycle to control for any circadian variations. Experimental procedures were in compliance with the National Institute of Health guidelines and approved by the University of Southern California and University of Kansas Animal Use Committees.

### ARS Regimen

All groups received a total of 4h of food and water deprivation prior to behavioral testing to control for any appetite-related effects. WT and MAO-A KO mice were divided into three conditions: 1-h ARS; 4-h ARS; and non-restraint stress (NRS) groups. In the ARS groups, mice were restrained for 1- or 4-h in 50mL plastic conical tubes, with holes drilled at each end and on the sides to allow ventilation. NRS animals were briefly exposed to the conical tube and returned to their home cages for 4h. Rectal temperature was measured via a custom probe (Physitemp instruments) prior to and immediately following the stress regimen. The overall change in temperature (final temperature – initial temperature) was used as an index of stress-induced hyperthermia (Bouwknecht et al., 2007). Morphological and behavioral tests were performed on separate sets of stressed and non-stressed animals.

### Behavioral Tests

Mice (n = 59) were tested for anxiety-related behaviors using a battery of progressively stressful tasks in the listed order below. Each test was performed for 5min. Mice were briefly returned to their home cages in between paradigms. To maximize the behavioral analyses of stress, behavioral testing was conducted within a 45-min window immediately following ARS (Van der Heyden et al., 1997).

#### Open-Field

Analysis of the open-field behaviors was performed as previously described (Bortolato et al., 2013). Mice were placed in the center and their behavior was monitored for 5min. Analysis of locomotor activity was performed using Ethovision (Noldus Instruments). Behavioral measures included the distance travelled, meandering (overall turning of the animal), time spent in the center zone, and the percent distance travelled in the center quadrant (calculated as percentage of total distance travelled by the mouse).

#### Object Interaction

Object-related exploration was performed as previously described (Godar et al., 2011). Mice were placed in a corner, facing the center, and at equal distance from two identical objects for 5min. The start position was rotated and counterbalanced for each genotype and condition throughout the tests. Exploratory approaches and duration were analyzed. Exploration was defined as sniffing or touching objects with the snout; climbing or sitting on the object was not considered exploration.

#### Elevated Plus-Maze

Anxiety-related behaviors were studied as detailed elsewhere (Bortolato et al., 2009). Mice were individually placed on the central platform facing an open arm, and allowed to explore for 5min. All four paws inside an arm constituted an arm entry. Behavioral measures included: frequency and time spent in each partition; total head dips; and total stretch-attend postures (as defined in Bortolato et al., 2009).

### Golgi Histology and Dendritic Analyses

Adult male mice were overdosed with pentobarbital within 5min following ARS and transcardially perfused with saline. Brains were removed and processed for Golgi histology using a modification of Glaser and Van der Loos’ Golgi stain as previously described (Martin and Wellman, 2011).

Pyramidal neurons of the OFC and BLA were investigated in view of their central role in emotional reactivity, contextual appraisal, and adaptive learning (Walton et al., 2011; McEwen et al., 2012). Pyramidal neurons—defined by the presence of a distinct, single apical dendrite, two or more basilar dendritic trees extending from the base of the soma, and dendritic spines—in the OFC and BLA were reconstructed (Figure 2A and D). Neurons selected for reconstruction were located in the middle third of the section, did not have truncated branches, and were unobscured by neighboring neurons and glia, with dendrites that were easily discriminable by focusing through the depth of the tissue. Within the orbitofrontal cortex, 12 neurons per mouse, evenly distributed over superficial and deep layers and across hemispheres and meeting criteria for reconstruction, were randomly selected and reconstructed. Following the same procedure, eight neurons per mouse from the basolateral amygdala, evenly distributed across hemispheres, were also reconstructed. The orbitofrontal cortex and basolateral amygdala were readily identifiable using standard cytoarchitectural and morphological criteria (Paxinos and Franklin, 2001).

Neurons were drawn at a final magnification of 600× and dendritic morphology was quantified in 3 dimensions using a computer-based neuron tracing system (Neurolucida; MBF Bioscience). Differences in the amount and location of dendritic material were quantified using a three-dimensional version of a Sholl analysis.

### Statistical Analyses

Normality and homoscedasticity of data distribution were verified by using Kolmogorov-Smirnov and Bartlett’s tests. Statistical analyses on parametric data were performed with one-way or two-way analyses of variance (ANOVAs), followed by Newman-Keuls test for post hoc comparisons. The significance threshold was set at 0.05. Morphological data were compared across groups using three-way repeated-measures ANOVA (genotype × stress condition × distance from soma). Significant main effects were followed up using two-way repeated measures ANOVAs, comparing either stress effects within genotype or effect of genotype in unstressed mice; significant interactions were followed up with planned comparisons consisting of two-group F-tests done within the context of the overall ANOVA (Hays, 1994).

## Results

### Acute Stress Induces Neophobic and Anxiety-Like Behaviors

**Figure 1.**
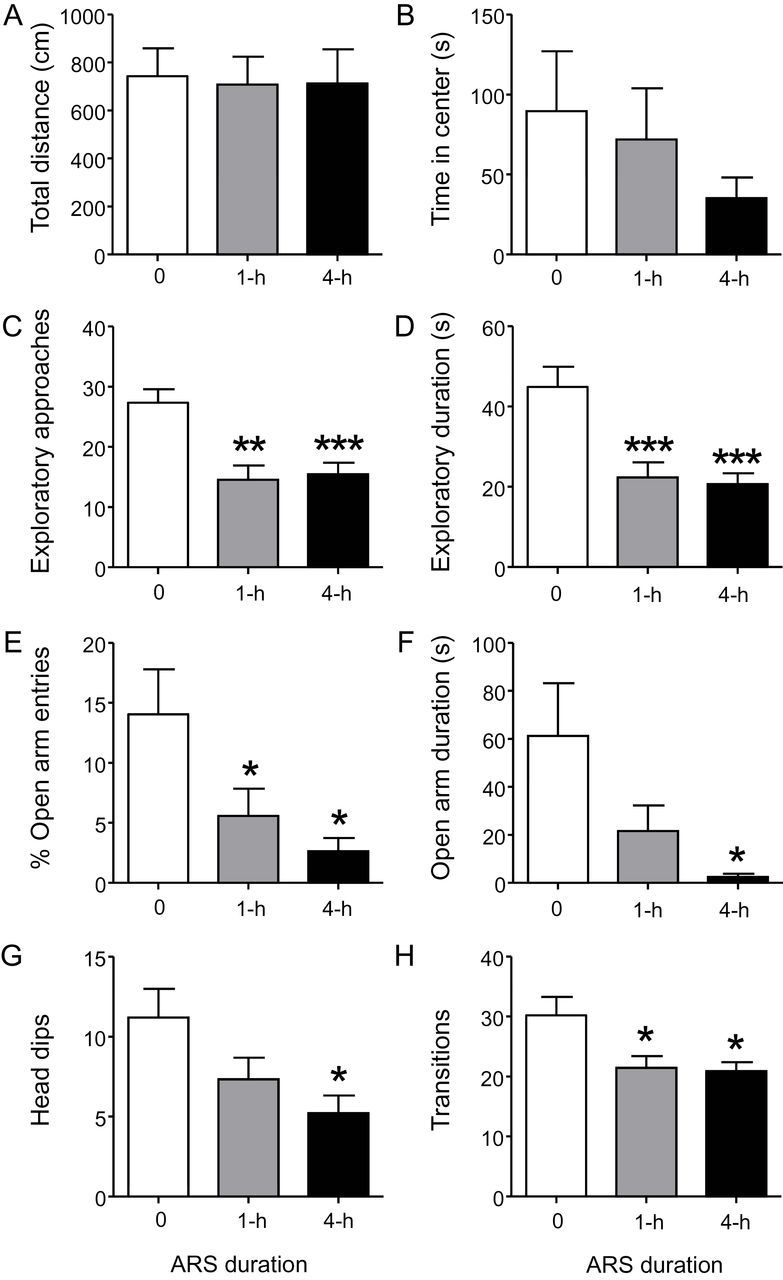
Acute restraint stress (ARS) significantly enhances anxiety-like behaviors. ARS did not alter (A) total distance traveled or (B) time spent in the center in the open field paradigm. In contrast, this manipulation markedly reduced (C) exploratory approaches and (D) duration towards novel objects. Similarly, in the elevated plus-maze, ARS produced a significant reduction in the (E) percent entries and (F) time spent in the open arms, two well-validated indices for anxiety-related behavior. Moreover, ARS resulted in significantly fewer (G) head dips and (H) overall number of transitions in the elevated plus-maze. Values displayed as means ± standard error of the mean. *p < 0.05, **p < 0.01, and ***p < 0.001 compared to non-stressed animals. Results are shown for 1-h ARS; 4-h ARS; and non-restraint stress (0) groups.

We first examined the impact of different durations of ARS on spontaneous behaviors (Table 1). Animals subjected to ARS did not exhibit any locomotor alterations in the open-field paradigm, including total distance [Figure 1B; *F*(2,25) = 0.02, NS], time spent in the center [Figure 1A; *H*(2) = 0.29, NS], percent activity in the center [NRS: 31.4±36.8; 1-h ARS: 24.9±29.0; 4-h ARS: 15.2±14.9; *F*(2,25) = 0.68, NS], or meandering [NRS: 151.9±110.8; 1-h ARS: 110.3±37.2; 4-h ARS: 129.9±63.5; *H*(2) = 0.14, NS]. Conversely, ARS elicited a marked reduction in novel object exploratory approaches [Figure 1C; *F*(2,26) = 11.24; *p* < 0.001] and duration [Figure 1D; *F*(2,26) = 11.21; *p* < 0.001] in WT mice (*p*s < 0.001; NRS WT vs 1-h and 4-h ARS).

**Table 1.** Acute restraint stress elicits a significantly lower body temperature response in MAO-A KO mutants than WT counterparts.

	**∆ Temperature**	
	1-hr ARS	4-hr ARS
**WT**	0.76±0.78	0.19±0.79

	1-hr ARS	4-hr ARS
**MAO-A KO**	N/A	–0.31 ± 0.66*

Values displayed as means ± SD. *p<0.05 compared to WT mice subjected to 4-h ARS.

In the elevated plus-maze, ARS significantly reduced the percent of open-arm [Figure 1E; *F*(2,25) = 4.94; *p* < 0.05] and closed arm-entries [NRS: 35.6±11.4; 1-h ARS: 43.4±6.4; 4-h ARS: 46.8±2.8; *F*(2,25) = 4.59; *p* < 0.05], as well as the time spent in the open-[Figure 1F; *F*(2,25) = 4.13; *p* < 0.05] and closed-arms [NRS: 150.1±70.4; 1-h ARS: 215.2±46.3; 4-h ARS: 246.9±42.4; *F*(2,25) = 6.86; *p* < 0.01], but not in the center [NRS: 85.6±44.6; 1-h ARS: 58.3±30.0; 4-h ARS: 47.1±42.6; *F*(2,25) = 2.12, NS]. Post hoc analyses revealed that 1-h ARS produced a reduction in the percent of open-arm entries (*p* < 0.05) and an increase in the closed-arm duration time (*p* < 0.05) in comparison to NRS WT mice. Open-arm duration and percent of closed-arm entries were reduced and increased, respectively; neither comparison, however, reached statistical significance (open-arm duration: *p* < 0.08; percent closed-arm entries: *p* < 0.06). In contrast, animals exposed to 4-h ARS displayed a more robust reduction in the percent of open-arm entries and open-arm duration (*p*s < 0.05), and significantly higher percent of closed-arm entries (*p* < 0.05) and closed-arm duration (*p* < 0.01) compared to NRS counterparts. ARS also produced a significant reduction in the number of arm transitions [Figure 3G; *F*(2,25) = 5.02; *p* < 0.05] and head dips [Figure 3H; *F*(2,25) = 4.32; *p* < 0.05], but not stretch-attend postures [NRS: 10.1±6.0; 1-h ARS: 9.4±3.1; 4-h ARS: 8.0±4.1; *F*(2,25) = 0.46, NS]. In particular, animals subjected to 1-h and 4-h ARS showed fewer arm transitions (*p*s < 0.05), but only 4-h ARS produced a statistically lower number of head dips (1-h: *p* < 0.08; 4-h: *p* < 0.05).

### ARS Produces Dendritic Remodeling of Pyramidal Neurons in the BLA and OFC in Mice

**Figure 2.**
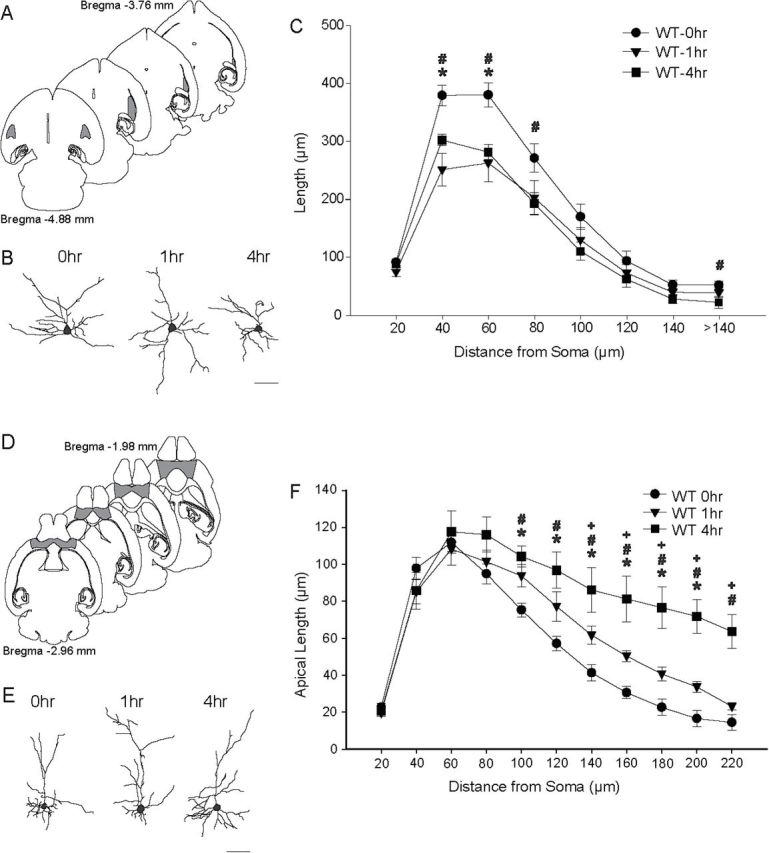
Acute restraint stress (ARS) remodels dendrites in the basolateral amygdala (BLA) and orbitofrontal cortex (OFC). (A) Schematic diagram of horizontal sections through the mouse brain. The shaded areas indicate portions of the BLA from which neurons were sampled. Coordinates indicate position in the dorsal-ventral axis relative to bregma (Paxinos and Franklin, 2001). (B) Reconstructions of representative pyramidal neurons from the BLAs in wildtype (WT) mice that were either unstressed (NRS; 0h) or underwent 1h or 4h of ARS. Neurons are near the mean for each group. Scale bar = 50 µm. (C) Mean length of BLA pyramidal neuron dendrites between 20-µm concentric spheres in mice that underwent 0h, 1h, or 4h of ARS. Dendritic retraction was evident immediately after either 1h or 4h of ARS. (D) Schematic diagram of horizontal sections through the mouse brain. The shaded areas indicate portions of the OFC from which neurons were sampled. (E) Reconstructions of representative pyramidal neurons from the OFCs in WT mice that underwent 0h, 1h, or 4h of ARS. (F) Mean length of OFC pyramidal neurons apical dendrites between 20-µm concentric spheres in WT mice that underwent 0h, 1h, or 4h of ARS. Either 1h or 4h ARS produced significant dendritic proliferation relative to NRS. For all graphs, *p < 0.05 for 0h vs. 1h; ^#^ *p* < 0.05 for 0h vs 4h; ^+^ *p* < 0.05 for 1h vs 4h.

ARS produced a very rapid remodeling of dendrites in the BLA [Figure 2B and C; *F*(2,22) = 5.26; *p* < 0.05], and this effect varied with distance from the soma [stress x distance from soma interaction, *F*(14,154) = 4.32; *p* < 0.05]. Specifically, 1-h ARS in WT mice significantly reduced dendritic length at 40 and 60 µm from the soma [*F*(1,17) = 24.33 and 10.53, respectively; *p* < 0.05]; furthermore, 4-h ARS produced more robust dendritic retractions, with significant differences relative to NRS WT mice at 40 through 80 µm and >140 µm [all *F*s(1,14) ≥ 4.86; *p* < 0.05]. WT mice subjected to 1-h and 4-h ARS did not significantly differ at any point in the dendritic arbor [all *F’s*(1,13) ≤ 3.17, NS].

ARS caused apical dendritic proliferation in the OFCs of WT mice [main effect of ARS: *F*(2,22) = 13.71; *p* < 0.05], an effect that varied with distance from the soma [Figure 2E and F; stress x distance from soma: *F*(22,242) = 4.94; *p* < 0.05]. In WT mice, 1-h ARS produced a modest but significant increase in dendritic material at distances of 100 through 200 µm [Figure 2F; all *F’s*(1,17) ≥ 5.51; *p* < 0.05; all other *F’s*(1,17) ≤ 3.11, NS]. Four hours of ARS dramatically increased apical dendritic material relative to both NRS and 1-h ARS WT mice. The 4-h ARS increased dendritic length compared to NRS mice at 100–220 µm from the soma [Figure 2F; all *F’s*(1,14) ≥ 17.38; *p* < 0.05; all other *F’s*(1,18) ≤ 4.38, NS]. Apical dendritic length was significantly increased by 4-h ARS compared to 1-h ARS at 140–220 µm from the soma [Figure 2F; all *F’s*(1,13) ≥ 4.56; *p* < 0.05; all other *F’s*(1,13) ≤ 2.43, NS]. No differences in basilar dendritic length were found [data not shown; main effect of stress, *F*(2,22) = 0.46, NS; stress × distance from soma interaction, *F*(14,154) = 0.98, NS).

To assess whether apical dendritic remodeling varied across layers within the OFC, a two-way ANOVA was performed, comparing total apical length across stress conditions and superficial version deep cortical layers. Overall, apical dendritic length did not vary across layers [F(1,22) = 0.69, NS]. However, ARS differentially affected apical dendritic morphology in superficial and deep cortical layers [data not shown; main effect of stress, F(2,22) = 12.10; *p* < 0.05; stress × cortical layer interaction, F(2,22) = 5.67; *p* < 0.05]. Follow-up planned comparisons demonstrated that 1-h ARS–induced apical dendritic proliferation was restricted to deep layers [deep: F(1,17) = 13.13; *p* < 0.05; superficial: F(1,17) = 0.91, NS], while 4-h ARS produced apical proliferation in both superficial and deep layers of OFC [F(1,14) = 6.54 and 41.33, respectively; both *p* < 0.05]. Apical dendritic length was significantly increased by 4-h ARS compared to 1-h ARS in the deep layers only [superficial, F(1,13) = 2.53, NS; deep, F(1,13) = 5.30; *p* < 0.05].

### MAO-A KO Mice may be Resistant to the Effects of ARS

**Figure 3.**
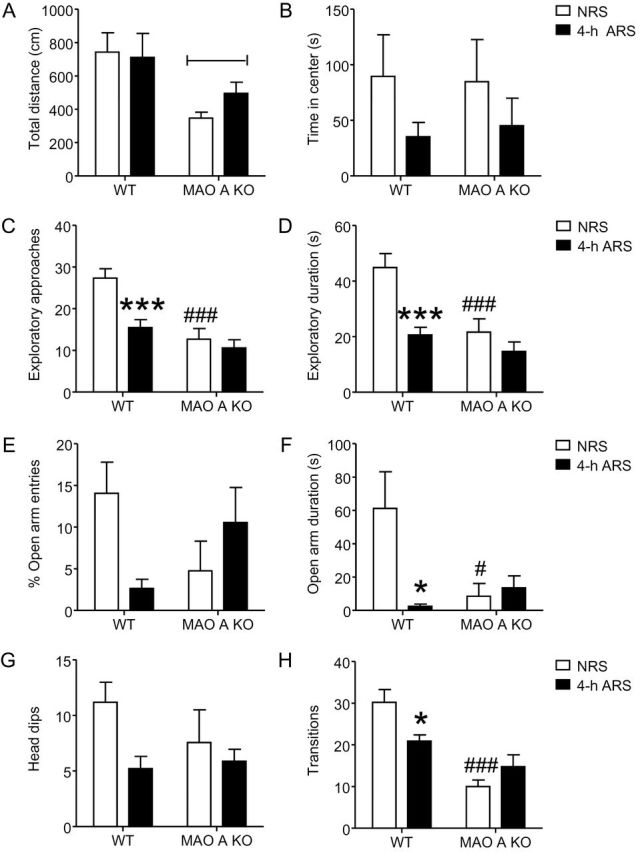
Acute restraint stress (ARS) does not alter emotional behaviors in MAO-A-deficient mice. (A–B) ARS did not affect locomotor activity in either genotype. (C–D) Although ARS increased neophobia in WT animals, MAO-A KO animals were unaffected. (E–H) Similarly, ARS enhanced anxiety-like behaviors in WT animals, but not in MAO-A mutant littermates. Values displayed as mean ± SEM. *p<0.05 and ***p<0.001 in WT mice exposed to 4-h ARS compared to non-stressed (NRS) WT controls. #*p*<0.05 and ###*p*<0.001 in NRS MAO-A KO mice compared to NRS WT mice. ΦΦ*p*<0.01 compared to WT animals.

To examine the role of MAO-A in mediating early stress responses, we compared the behavioral and morphological effects of WT and MAO-A KO mice left unstressed (NRS) or subjected to 4-h ARS. We limited our analyses of early stress responses to the 4-h exposure, since this duration elicited the most robust effect on both behavior and morphology in WT animals. MAO-A KO mice subjected to 4-h ARS displayed a significant decrease in hyperthermia [Table 1; *F*(1,36) = 4.50; *p* < 0.05] compared to WT mice exposed to the same condition. In the open field paradigm, MAO-A KO mice showed a reduction in total distance covered [Figure 3A; genotype: *F*(1,36) = 9.81; *p* < 0.01] and an increase in meandering [WT NRS: 151.9±110.8; 4-h ARS: 129.9±63.5; MAO-A KO NRS: 251.7±141.1; 4-h ARS: 225.5±195.4; genotype: *F*(1,36) = 4.39; *p* < 0.05] that were genotype-specific. Conversely, no significant genotype x stress interactions were found in total distance [Figure 3A; *F*(1,36) = 0.84, NS], time in center [Figure 3B; *F*(1,36) = 0.06, NS], percent activity in center [WT NRS: 31.4±36.8; 4-h ARS: 15.2±14.9; MAO-A KO NRS: 32.3±36.5; 4-h ARS: 19.6±27.9; *F*(1,36) = 0.03, NS], or in meandering [*F*(1,36) = 0.00, NS].

ARS induced significant alterations in exploratory approaches [Figure 3C; genotype x ARS: *F*(1,35) = 5.08; *p* < 0.05] and duration [Figure 3D; genotype x ARS: *F*(1,35) = 4.23; *p* < 0.05] towards novel objects. Post hoc analyses showed that NRS MAO-A–deficient mice displayed a marked decrease in both exploratory frequency (*p* < 0.001) and duration (*p* < 0.001) compared to NRS WT animals. Similarly, 4-h ARS induced a decrease in exploratory approaches (*p* < 0.001) and duration (*p* < 0.001) in WT, but not in MAO-A KO mice, compared to their NRS counterparts.

In the elevated plus-maze, significant genotype x stress interactions were identified for percent open-arm [Figure 3E; genotype x ARS: *F*(1,30) = 7.13; *p* < 0.05] and closed-arm [WT NRS: 35.6±11.4; 4-h ARS: 46.8±2.8; MAO-A KO NRS: 43.4±14.2; 4-h ARS: 38.1±12.0; genotype x ARS: *F*(1, 30) = 4.35; *p* < 0.05] entries; however, no individual group differences were found by post hoc testing. In contrast, a genotype x stress interaction was detected for the time spent in the open-arms [Figure 3F; genotype x ARS: *F*(1,30) = 5.20; *p* < 0.05], but not in closed-arms [WT NRS: 150.1±70.4; 4-h ARS: 246.9±42.4; MAO-A KO NRS: 169.0±103.2; 4-h ARS: 213.1±87.6; genotype x treatment: *F*(1,30) = 0.86, NS] or center [WT NRS: 85.6±44.6; 4-h ARS: 47.1±42.6; MAO-A KO NRS: 119.7±93.7; 4-h ARS: 71.1±75.1; genotype x ARS: *F*(1,30) = 0.05, NS]. Post hoc analyses revealed that NRS WT mice spent more time in the open arms than both NRS MAO-A KO mice and WT mice subjected to 4-h ARS (*p* < 0.05). Similarly, both genotype and stress had a significant effect on the number of transitions [Figure 3G; genotype x ARS: *F*(1,30) = 7.78; *p* < 0.01], where NRS WT mice engaged in more arm transitions than either NRS MAO-A–deficient mice (*p* < 0.001) or WT animals exposed to 4-h ARS (*p* < 0.05). No differences in genotype x stress were found for head dips [Figure 3H; genotype x ARS: *F*(1,30) = 1.43, NS] or stretch-attend postures [WT NRS: 10.1±6.0; 4-h ARS: 8.0±4.1; MAO-A KO NRS: 8.3±5.0; 4-h ARS: 10.7±5.9; genotype x ARS: *F*(1,30) = 1.28, NS].

### MAO-A KO Mice Exhibit Reduced Dendritic Length in BLA Pyramidal Neurons

**Figure 4.**
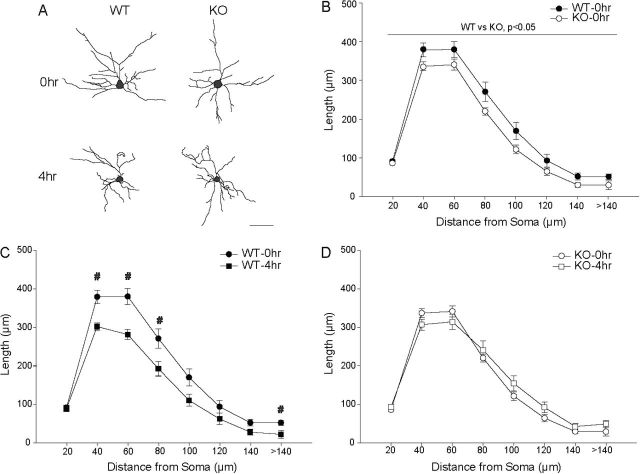
Acute restraint stress (ARS) does not produces dendritic retraction in basolateral amygdala of MAO-A KO mice. (A) Reconstructions of representative pyramidal neurons from basolateral amygdala in WT versus MAO-A knockout (KO) mice that were either unstressed (NRS, 0h) or underwent 4h of ARS. Neurons are near the mean for each group. Scale bar = 50 µm. (B) Mean length of dendrites between 20-µm concentric spheres in WT versus KO mice. KO mice show dendritic retraction relative to WT. (C) Mean length of dendrites between 20-µm concentric spheres in WT mice that underwent 0h or 4h of ARS. Four-hour ARS produced significant apical dendritic retraction relative to NRS. (D) Mean length of apical dendrites between 20-µm concentric spheres in KO mice that underwent 0h or 4h of ARS. In KO mice, 4-h ARS did not significantly alter dendritic morphology. For all graphs, #p<0.05 for 0hr vs 4hr.

Although there was no main effect of genotype [*F*(1,29) = 0.07;NS], we found a significant genotype x stress interaction [*F*(2,29) = 7.30; *p* < 0.05] on dendritic length. Two-way repeated measures ANOVA (genotype × distance from soma) in NRS WT versus MAO-A KO mice demonstrated that across the dendritic arbor, dendritic length was significantly decreased in MAO-A KO mice [Figure 4A and B; genotype: *F*(1,18) = 5.18; *p* < 0.05; genotype × distance from soma: *F*(7,126) = 1.17, NS].

### ARS Does Not Produce Dendritic Remodeling in the BLA Pyramidal Neurons of MAO-A KO Mice

Although there was no main effect of stress [*F*(1,29) = 3.81;*p* < 0.05], alterations in dendritic length varied with genotype and distance from the soma [Figure 4A, C, and D; stress x distance from soma: *F*(7,203) = 5.49; *p* < 0.05; genotype x distance from soma: *F*(7,203) = 0.41, NS]. No three-way interaction was present [stress x genotype x distance from soma: *F*(7,203) = 1.86, NS]. Whereas ARS significantly reduced dendritic length in the BLAs of WT mice (see above), MAO-A KO mice that underwent 4-h ARS did not differ from NRS KO at any point in the arbor [all *F*s(1,13) ≤ 2.96, NS].

### MAO-A KO Mice Exhibit Increased Apical Dendritic Length in OFCs

**Figure 5.**
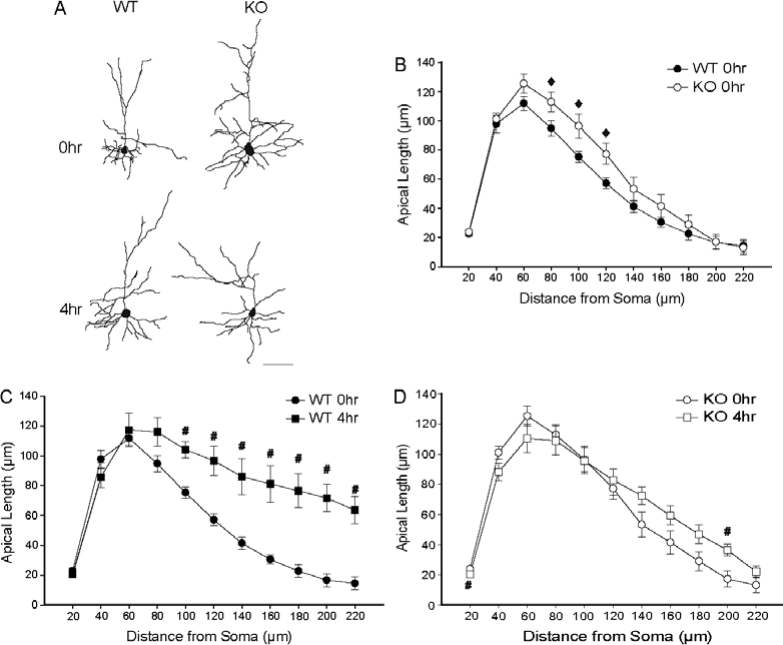
Acute restraint stress (ARS) does not produces dendritic proliferation in the orbitofrontal cortices of monoamine oxidase A (MAO-A) knockout (KO) mice. (A) Reconstructions of representative pyramidal neurons from OFCs in wildtype (WT) versus MAO-A KO mice that were either unstressed (NRS, 0h) or underwent 4h of ARS. Neurons are near the mean for each group. Scale bar = 50 µm. (B) Mean length of apical dendrites between 20-µm concentric spheres in WT versus KO mice. KO mice show apical dendritic proliferation relative to WT. (C) Mean length of apical dendrites between 20-µm concentric spheres in WT mice that underwent 0h or 4h of ARS. Four-hour ARS stress produced significant apical dendritic proliferation relative to NRS. (D) Mean length of apical dendrites between 20-µm concentric spheres in KO mice that underwent 0h, 1h, or 4h of ARS. ARS stress produced minimal apical dendritic proliferation in KO mice. For all graphs, ^♦^ *p* < 0.05 for 0hr WT vs 0hr KO; ^#^ *p* < 0.05 for 0hr vs 4hr.

We found no main effect of genotype [*F*(1,29) = 0.24;NS], but significant interactions of stress and genotype [*F*(1,29) = 5.75, *p* < 0.05] and genotype and distance from soma [*F*(11,319) = 2.40; *p* < 0.05] in the amount and distribution of apical dendritic material. Planned comparisons between NRS WT and KO mice demonstrated that dendritic length was significantly increased at 80–120 µm from the soma in MAO-A KO relative to WT mice [Figure 5A and B; for 80–120 µm, all *F’s*(1,18) ≥ 4.41; *p* < 0.05; all other *F’s*(1,18) ≤ 2.67, NS].

### ARS Does Not Alter Dendritic Morphology in OFCs of MAO-A KO Mice

ARS significantly altered apical dendritic length [*F*(2,29) = 14.60; *p* < 0.05], an effect that varied across genotypes [stress x genotype: *F*(2,29) = 5.75; *p* < 0.05] and distance from the soma [Figure 2D and E; stress x distance from soma: *F*(11,319) = 8.34; *p* < 0.05]. No three-way interaction was present [stress x genotype x distance from soma: *F*(11,319) = 1.40, NS]. In WT mice, four hours of ARS dramatically increased apical dendritic material relative to NRS mice (Figure 5A and C). However, apical dendritic morphology was essentially unaffected in MAO-A KO mice: stress produced a small but significant increase in apical dendritic length at only one point in the arbor, 200 µm from the soma [Figure 5A and D; *F*(1,15) = 7.39; *p* < 0.05; all other *F’s*(1,15) ≤ 3.18, NS].

No differences in basilar dendritic length were found [data not shown; genotype: *F*(1,29) = 0.66, NS; ARS: *F*(1,29) = 0.10, NS; stress x genotype: *F*(1,29) = 1.56, NS; genotype x distance from soma: *F*(7,203) = 1.36; genotype x stress x distance from soma; *F*(7,208) = 0.70, NS]. Although a stress × distance from soma interaction was present [*F*(7,203) = 3.06; *p* < 0.05], planned comparisons collapsed across genotypes failed to reveal significant differences at any distance from the soma [all *F*s(1,31) ≤ 3.17, NS].

## Discussion

The results of this study showed that, in WT mice, 1- and 4-h ARS resulted in a progressive enhancement of neophobic and anxiety-like behaviors, which was accompanied by marked dendritic proliferation in the OFC and dendritic retraction in the BLA. These findings provide the first documentation that ARS leads to fast dendritic remodeling, and support the possibility that the length and number of dendritic branches of pyramidal neurons may undergo rapid modifications in response to salient environmental inputs.

Acute stress is known to elicit anxiety and defensive responses (Rodgers and Cole, 1993; Barros et al., 2008). Accordingly, we found that ARS reduced two well-established indices of anxiolysis in animal models: namely, novel object exploration and open-arm duration in the elevated plus-maze (Rodgers and Cole, 1994; Hughes, 2007). Notably, the enhancement in anxiety-related behaviors corresponded with progressive changes in OFC dendritic length and was independent of locomotor activity. This rapid dendritic remodeling in this region may serve as a cellular correlate of the enhanced neophobic and anxiety-like responses; indeed, anxiety has been related to variations in OFC thickness (Blackmon et al., 2011; Kühn et al., 2011). Given that the geometrical characteristics of dendritic arbor (including branching patterns, distribution, and overall shape) determine many functional properties of neurons (Mainen and Sejnowski, 1996; Koch and Segev, 2000; Grudt and Perl, 2002), the stress-induced dendritic changes may contribute to stress-induced alterations in OFC- and BLA-mediated behaviors, such as behavioral flexibility, decision-making, and adaptive responses to contextual cues, as well as emotional reactivity and control. The morphological alterations in these two regions may reflect the top-down control of the prefrontal cortex over amygdala reactivity in stress resilience (Franklin et al., 2012; Wheelock et al., 2014). In particular, the dendritic remodeling in the OFC may signify specific adaptive changes related to the appraisal of stress controllability and threat, given the role of this brain area in these functions (Kalin et al., 2007; Ohira et al., 2008; Gold et al., 2014).

Our morphological findings in adult mice complement previous reports of rapid changes in dendritic remodeling during development and neuronal maturation (Dailey and Smith, 1996; Kaethner and Stuermer, 1997; Wu et al., 1999). In addition, these results expand on previous reports showing that chronic stress induces dendritic proliferation in the OFC (Liston et al., 2006) and impairs decision-making strategies (Dias-Ferreira et al., 2009).

The observed dendritic changes in the OFC were more prevalent in the distal portion of the apical dendrites; this distinct pattern of dendritic remodeling may reflect differences in stress-induced alterations of specific inputs to the dendritic arbors. For instance, 5-HT2A receptor-mediated excitatory inputs are localized on the apical dendrites of neocortical pyramidal neurons (Aghajanian and Marek, 1997; Liu and Aghajanian, 2008), and dopaminergic neurons densely innervate the superficial layers of the OFC (e.g. Goldman-Rakic et al., 1990). Previous studies have shown that apical dendrites of cortical pyramidal cells form thalamo-cortical and cortico-cortical connections involved in the processing of sensory feedback loops (Thomson and Lamy, 2007; Rubio-Garrido et al., 2009). The application of stress may lead to an enhancement of glutamate-mediated sensory input onto OFC circuits to better coordinate decision-making processes (Moghaddam, 2002). Based on this background, it is possible that the specific increase in apical, but not basilar, dendrites of OFC neurons may serve as an adaptive mechanism that attunes the neuron to gate information from sensory feedback circuits.

The robust dendritic retraction of BLA pyramidal neurons induced by ARS is consistent with a recent study showing dendritic retraction in BLA pyramidal neurons 3 days after an acute elevated platform stressor (Maroun et al., 2013); however, this finding conflicts with a previous report that failed to detect alterations in BLA dendritic morphology after acute immobilization stress (Mitra et al., 2005). This apparent discrepancy may be explained by the use of different stressors, given the divergent sensitivity of BLA morphology to specific chronic stressors (Vyas et al., 2002). In addition, it should be noted that, in the present study, dendritic morphology was examined immediately after ARS, whereas Mitra and colleagues (2005) assessed dendritic morphology after a delay of 1 to 10 days post-stress. It is possible that there may be a dynamic, non-linear time-course of stress-induced changes in the BLA, with neurons undergoing initial dendritic retraction (during the response to stress or threat) followed by recovery (during extinction; Heinrichs et al., 2013). Accordingly, this recovery period may depend on stressor intensity and/or duration, in which longer or repeated stressors may inhibit this process. Such a pattern is consistent with the dendritic remodeling observed after experimental manipulations that alter neuronal inputs, such as deafferentation, which typically results in initial dendritic retraction followed by proliferation (e.g. Matthews and Powell, 1962; Caceres and Steward, 1983). Although the present study did not include analyses of the changes in remodeling at different time points following ARS, future investigations are warranted to analyze whether (and within what timeframe) the observed changes may be reversible. Similarly, future research is needed to test whether the morphological changes of the dendritic arbor of OFC and BLA neurons can be compounded by longer durations of restraint stress as well as other factors (including stressor intensity, controllability, etc.).

The dendritic retraction in the BLA was primarily confined to proximal areas. Proximal dendrites of BLA pyramidal cells are innervated by inhibitory interneurons and are more likely to impact neuronal firing processes (Maren and Quirk, 2004). In contrast, the density of excitatory synapses increases as one proceeds towards the distal dendritic shaft (Muller et al., 2006, 2007). Studies have shown that unconditioned aversive stress inhibits BLA-targeted interneurons and likely results in disinhibition of proximal pyramidal cell excitatory input, favoring synaptic plasticity and ultimately affecting gating of emotional learning (Wolff et al., 2014). Thus, the reduction in dendritic length may be an active process targeting the dampening of emotional responses by increasing cortically-driven excitatory signals in order to facilitate stress resilience (Franklin et al., 2012). Indeed, imaging studies have shown that stress exposure therapy reduces amygdalar responses to subsequent stressors, which may signify a gradual adaptation to emotional stimuli (Nechvatal and Lyons, 2013).

Of note, restraint stress has been proposed as one of the most severe paradigms to elicit manifestations reminiscent of post-traumatic stress disorder (PTSD; Liston et al., 2006). The OFC has also been implicated in PTSD, where it functions in the processing of fear and the extinction of emotional memory (Newport and Nemeroff, 2000). Thus, the stress-induced increased arborization in the OFC may contribute to the heightened emotional responses to fear or threats following stress exposure (Newport and Nemeroff, 2000). Interestingly, recent neuroimaging data indicate reduced amygdala volume in veterans with PTSD relative to combat-exposed controls (Rogers et al., 2009; Morey et al., 2012). Further, consistent with dendritic retraction in the BLA, trauma exposure in veterans was found to be associated with smaller amygdala volumes, regardless of PTSD diagnosis, with the severity of exposure correlating negatively with amygdala volume (Morey et al., 2012).

Another important finding of our study was that MAO-A KO mice failed to exhibit similar neurobehavioral alterations as WT littermates in response to ARS. This finding is in line with previous lines of evidence indicating that MAO-A activation is instrumental for the enactment of stress response. For example, stress has been shown to facilitate the transient release and metabolism of monoamines in various brain areas, including the cortex and amygdala (Flugge et al., 2004; Joels and Baram, 2009). Several lines of evidence have shown that MAO-A inhibitors reduce anxiety and neophobia (Caille et al., 1996; de Angelis, 1996; Eroglu and Guven, 1998; Steckler et al., 2001); furthermore, the antidepressant effects of these drugs have been shown to reflect their ability to increase the resilience to acute stress (Miura et al., 1996; Ferigolo et al., 1998; Cryan et al., 2005).

Previous investigations have shown that low levels of MAO-A expression and/or function have been associated with blunted response of the hypothalamic-pituitary-adrenal axis to stress (Reul et al., 1994; Brummett et al., 2008). Furthermore, MAO-A deficiency results in blunted neuroendocrine responses to major stressors in humans and rodents (Brunner et al., 1993; Cases et al., 1995; Popova et al., 2006; Godar et al., 2011; Bortolato et al., 2013), as well as reduced extinction of aversive stimuli, a primary symptom in PTSD (Kim et al., 1997; Parsons and Ressler, 2013).

These findings may suggest that MAO-A deficiency leads to a generalized resistance to the neurobehavioral effects of ARS. However, it should be noted that, in line with our previous findings (Bortolato et al., 2011), NRS MAO-A KO mice display significantly higher dendritic arborization in the OFC and dendritic retraction in the BLA compared to their WT counterparts. Interestingly, the progressive ARS-induced alterations in dendritic morphology in WT mice were remarkably similar to NRS MAO-A KO animals. Thus, the baseline phenotype of MAO-A KO mice may correspond to a stressed state in WT littermates, thereby masking potential exacerbations due to ARS. Indeed, our previous analyses showed that, while MAO-A KO mice do not display appropriate reactions to stress, they exhibit exaggerated defensive responses to innocuous and neutral environmental stimuli, including novel environmental cues (Godar et al., 2011).

The molecular involvement of MAO-A in the rapid dendritic remodeling remains unclear, but may involve a role of MAO-A in the modulation of microtubule dynamics, the core process underpinning changes in dendritic morphology (Gardiner et al., 2011). Specifically, previous data have shown that, in mice, modifications of dendritic length and spine density are orchestrated by the neurotrophin brain-derived neurotrophic factor (BDNF; Horch, 1999; Tolwani et al., 2002; Jin et al., 2003; Kellner et al., 2014) through regulation of microtubule stability (Jaworski et al., 2009). Interestingly, MAO-A has been shown to modulate BDNF activity and function in a region-dependent fashion (Balu et al., 2008; Fortunato et al., 2010).

In addition to BDNF, other factors may participate in the monoaminergic regulation of neuroplastic processes. Monoamine neurotransmitters have been shown to influence the subunit composition and function of glutamate N-methyl-D-aspartate receptors (NMDAR; Bortolato et al., 2012), critical regulators of stress-induced plasticity (Ziegler et al., 2005; Martin and Wellman, 2011), and informational salience (Seeburg et al., 1995; Phillips et al., 2010). For instance, NMDAR activity, and specifically the NR2B subunit, has been recently implicated in the modulation of dendritogenesis (Bustos et al., 2014). In line with this possibility, MAO-A KO mice exhibit profound disruptions in the biophysical and functional properties of NMDARs in the prefrontal cortex (Bortolato et al., 2012), which likely underpin their emotional (Kim et al., 1997) and contextual processing impairments (Godar et al., 2011). Another intriguing mechanism that may be involved in the influence of MAO-A on neuroplastic phenomena may be mediated by tissue plasminogen activator (tPA). This extracellular protease, which has been shown to play a key role in the modification of spine density in response to stress (Bennur et al., 2007), controls NMDAR function (Nicole et al., 2001), as well as the post-transcriptional maturation of BDNF (Pang et al., 2004). Of note, preliminary research has shown that tPA modulates dopamine and serotonin neurotransmission (Centonze et al., 2002; Pothakos et al., 2010). This background highlights the need for future studies to evaluate the relevance of BDNF, NMDAR, tPA, and other molecular determinants in the role of MAO-A and its neurotransmitter substrates in the regulation of acute stress response.

Irrespective of the specific mechanisms mediating the role of MAO-A in the ontogeny of the neurobehavioral changes induced by ARS, our data suggest that this enzyme is required for rapid alterations in OFC and BLA dendritic remodeling in response to acute stress, which may in turn contribute to the enactment of defensive and neophobic behaviors. In conclusion, our findings further underscore the importance of MAO-A and monoamine metabolism in the modulation of the morphological and behavioral adaptive responses to stress.

## Statement of Interest

All authors declare no financial or other conflicts of interest.
